# Complete Genome Sequence of the Newly Developed *Lactobacillus acidophilus* Strain With Improved Thermal Adaptability

**DOI:** 10.3389/fmicb.2021.697351

**Published:** 2021-09-24

**Authors:** Soomin Jeon, Hyaekang Kim, Youngseok Choi, Seoae Cho, Minseok Seo, Heebal Kim

**Affiliations:** ^1^Department of Agricultural Biotechnology, Research Institute of Agriculture and Life Sciences, Seoul National University, Seoul, South Korea; ^2^eGnome, Inc., Seoul, South Korea; ^3^Department of Computer Convergence Software, Korea University, Sejong, South Korea; ^4^Interdisciplinary Program in Bioinformatics, Seoul National University, Seoul, South Korea

**Keywords:** adaptive laboratory evolution (ALE), *Lactobacillus acidophilus*, heat resistance, whole-genome sequencing, bacterial evolution

## Abstract

*Lactobacillus acidophilus* (*L. acidophilus*) is a representative probiotic and is widely used in many industrial products for its beneficial effects on human and animal health. This bacterium is exposed to harsh environments such as high temperatures for manufacturing industrial products, but cell yield under high temperatures is relatively low. To resolve this issue, we developed a new *L. acidophilus* strain with improved heat resistance while retaining the existing beneficial properties through the adaptive laboratory evolution (ALE) method. The newly developed strain, *L. acidophilus* EG008, has improved the existing limit of thermal resistance from 65°C to 75°C. Furthermore, we performed whole-genome sequencing and comparative genome analysis of wild-type and EG008 strains to unravel the molecular mechanism of improved heat resistance. Interestingly, only two single-nucleotide polymorphisms (SNPs) were different compared to the *L. acidophilus* wild-type. We identified that one of these SNPs is a non-synonymous SNP capable of altering the structure of MurD protein through the 435th amino acid change from serine to threonine. We believe that these results will directly contribute to any industrial field where *L. acidophilus* is applied. In addition, these results make a step forward in understanding the molecular mechanisms of lactic acid bacteria evolution under extreme conditions.

## Introduction

Lactic acid bacteria (LAB) is a gram-positive bacteria that produces lactic acid as a fermentation product (Makarova et al., 2006; Zheng et al., 2020). Since LAB has been mainly applied in dairy products or fermented foods for humans and animals, it has been domesticated toward profitable ends (Steensels et al., 2019). For example, these bacteria are highly resistant to acids and bile salts, making them widely used for industrial purposes such as food manufacturing (Menconi et al., 2014). Functionally, they play a beneficial role in inhibiting the growth of pathogens by producing antimicrobial compounds such as lactic acid, hydrogen peroxide, and bacteriocin (Mohankumar and Murugalatha, 2011).

*Lactobacillus acidophilus* (*L. acidophilus*) is a representative LAB species that has been well studied in its physiology and functionality. The beneficial health effects of *L. acidophilus* have been shown in studies of various diseases such as innate immunity (Klein et al., 2008; Foysal et al., 2020), inflammatory bowel disease (Peran et al., 2007; Park et al., 2018), and colon cancer (Zhuo et al., 2019). In addition, as scientific proof comes to light about various effects such as skin wrinkle improvement (Chahuki et al., 2019), skin moisturization (Im et al., 2018), and vaginal cleansing (Bertuccini et al., 2017), the scope of utilizing *L. acidophilus* in the industrial field is gradually expanding. Based on the scientific evidence of these health benefits, *L. acidophilus* holds an important position in the probiotic market, and a variety of commercial strains have been discovered such as *L. acidophilus* NCFM (Altermann et al., 2005), *L. acidophilus* LA-1 and LA-5 (Schillinger et al., 2003; Matijašić et al., 2016), and *L. acidophilus* DDS-1 (Dash, 2004). *L. acidophilus* is known to have the same high acid, bile salt, and osmotic resistance as the common LAB (Hutkins et al., 1987; Chou and Weimer, 1999).

When LAB are used for manufacturing industrial products, the bacterial strain is often exposed to extreme environments. Among various environmental factors, high temperature has a major influence on bacterial survival. For example, LAB are used as an animal feed additive, and they are usually manufactured in pellet form. Pellets are made by compressing ground feed and supplements including LAB by applying air above 80°C (Skoch et al., 1981). Some feed mills have compression temperatures that can reach 90°C to destroy feed-borne pathogens such as *Salmonella* (Jones and Richardson, 2004). After this process, the bacteria are useful to animals only if it survives in the pellet. Thermal and mechanical treatments have physiological and biological effects on living cells, such as denaturing proteins and altering enzymatic activity (Belhadj Slimen et al., 2016), which appears as a decrease in cell viability. Therefore, strains such as *Saccharomyces cerevisiae* and *Bacillus subtilis* with high survival rates under heat treatment are mainly used (Haldar et al., 2011; Nguyen et al., 2015). Among the lactic acid strains, *Enterococcus faecium* is mainly utilized (Boney et al., 2018). *L. acidophilus* has been suggested as an antibiotic alternative by improving growth performance and nutrient utilization in animal intestines, but their low thermal stability is a limitation for use as feed additives (Simon et al., 2005; Lan et al., 2017). Furthermore, in addition to direct heat treatment to LAB, a thermostable bacterial strain can help protect against heat-induced death or accidental thermal management defects during fermentation, thereby increasing cooling cost-effectiveness (Matsushita et al., 2016). Therefore, it is expected that improving the heat resistance of *L. acidophilus* will not only increase the industrial utility but also contribute to the expansion of the application range.

Encapsulation and heat pretreatment for heat-shock protein expression have been the main methods studied for improving the heat stress resistance of diverse bacterial strains, but these methods are not cost-effective (Xu et al., 2016; Chen et al., 2017). On the other hand, adaptive laboratory evolution (ALE) artificially stimulates natural evolution in a laboratory setting, making it relatively easy to improve the desired phenotype of targeted strain (Portnoy et al., 2011; Dragosits and Mattanovich, 2013). Research to increase the heat resistance of bacteria by applying the ALE method has been conducted in various species such as *Escherichia coli* (Riehle et al., 2003; Rudolph et al., 2010) and *E. faecium* (Min et al., 2020). Likewise, studies to improve the thermal resistance of LAB have been steadily carried out over the past 20 years, but most of the studies have only confirmed improved cell viability below 65°C. For example, survival rates of *Lacticaseibacillus paracasei* DPC1919, DPC2102, and DPC2013 strains were evaluated at 56–67.5°C (Jordan and Cogan, 1999). In another study, the thermal resistance of *L. acidophilus* LA1-1 was measured at 37–58°C (Kim et al., 2001). There have also been attempts to develop *L. acidophilus* EG008 strains using ALE such as *L. acidophilus* NCFM at 65°C (Kulkarni et al., 2018) and *Lactiplantibacillus plantarum* Lp 998 at 45–55°C (Ferrando et al., 2015).

Based on these rationales, the primary goal of this study is to develop a strain of *L. acidophilus* that can withstand conditions of 65°C or higher through the ALE method. The secondary goal is to minimize changes in the genetic background to maintain the functional advantages of the existing *L. acidophilus* strain as much as possible. We confirmed this through complete genome analysis using long-read sequencing.

## Materials and Methods

### Strain Identification and Bacterial Culture

A probiotic colony was isolated from a fermented dairy food. To identify bacterial species, 16S rRNA genes were sequenced by Macrogen Inc. (Seoul, South Korea). Sequencing reactions were performed in the DNA Engine Tetrad 2 Peltier Thermal Cycler (Bio-Rad, Hercules, CA, United States) using the ABI BigDye (R) Terminator v3.1 Cycle Sequencing Kit (Applied Biosystems, Beverly, MA, United States). Primers used for single-pass sequencing were as follows: forward primer 27F (5′-AGAGTTTGATCCTGGCTCAG-3′) and reverse primer 1492R (5′-GGTTACCTTGTTACGACTT-3′). To remove the unincorporated terminators and dNTPs, the fluorescent-labeled fragments were purified by the method that Applied Biosystems recommends. The samples were injected to electrophoresis in an ABI 3730xl DNA Analyzer (Applied Biosystems). Using BLAST, 16S rRNA sequences were compared with the NCBI database (Johnson et al., 2008). For bacterial culture, all strains were propagated statically in deMan Rogosa Sharpe broth (MRS broth; Difco Laboratories, Detroit, MI, United States) or on MRS agar [1.5% (wt/vol)] under aerobic condition at 37°C without shaking. Gram staining was performed using the BD BBL^TM^ Gram stain kit following the manufacturer’s protocol. For viable cell counting, serially diluted cultures were poured into the MRS plates and inoculated at 37°C for 48 h. The viability of the cell was counted in the colony-forming unit per milliliter (CFU/ml). Cell density was measured by the absorbance at 600 nm (OD_600_; optical density spectrophotometrically measured at 600-nm wavelength) using an OPTIZEN POP UV-visible spectrophotometer (KLAB, Daejeon, South Korea).

### Adaptive Laboratory Evolution and Screening a Thermal-Adapted Strain

**FIGURE 1:**
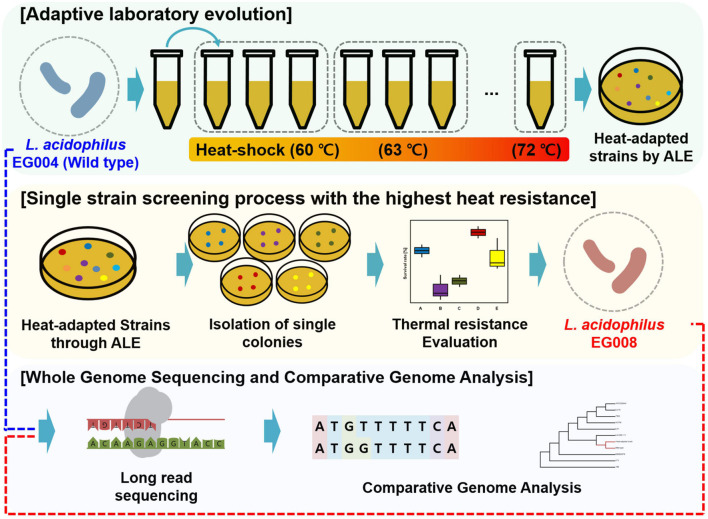
Schematic diagram of the study to develop *L. acidophilus* EG008 strain. This diagram shows the overall process for developing an EG008 strain of *L. acidophilus*. The first step is the thermal adaptation of *L. acidophilus* EG004 strains through adaptive laboratory evolution. The second step is the process of selecting the colonies that have the single strain with the highest heat resistance among the various strains that have been thermally adapted. The final step is the *in silico* analysis step to identify and compare the whole genome sequence of *L. acidophilus* EG004 and EG008 strains, respectively.

To induce the newly improved thermal tolerance of the strain, an ALE experiment at high temperature was applied. The development of a heat-adapted strain consisted of two steps: heat adaptation to the highest survival temperature and single strain selection with thermotolerance (Figure 1). *L. acidophilus* EG004 strain was used as wild-type, cultured for 16 h, and prepared for heat adaptation to the highest temperature. Ten microliters of cells were injected into 990 μl of pre-heated MRS broth at 60°C by heat block (ALB64; FINEPCR, Gyeonggi-do, South Korea). Heat treatment was applied for 1 min. After heat treatment, it was allowed to cool down at room temperature for 5 min and cultivated at 37°C for 24 h. After two iterations, an identical process was repeated with increased temperature by 3°C until incubation was impossible. All strains of each step were stored with 25% glycerol at −80°C. Next, we performed a single strain selection from the microbial population obtained through the final ALE treatment (A001F8-72). It was performed because the final result of ALE is presumed to be a group of individuals with random mutations rather than a single individual. The evaluation temperature was determined as the temperature above the critical point of EG004 strain. Five different colonies were picked from A001F8-72. A hundred microliters of cells were injected in 900 μl of pre-heated saline at 66°C by heat block, and cells were heated for 1 min. After cooling down, the diluted cells were plated on MRS agar to determine viable cell count. The cell survival ratio was expressed by dividing the viable cell count after heat stress by the initial viable cell count. The strain that had the most improved resistance to high temperature was named as “EG008” strain and used for the comparative analysis.

### Assessment of Phenotypical Changes

In order to assess improved thermal capacity, cell viability at 55–75°C was assessed. Cells in the mid-exponential phase were prepared to OD_600_ at 1.3, corresponding to approximately 1 × 10^9^ CFU/ml. A hundred microliters of cells were injected into 900 μl of pre-heated saline by heat block. After heat was applied for 1 min, cells were plated on MRS agar to quantify the viable cell count. Thermal resistance was presented as the survival ratio. To check other phenotypical changes induced by heat stress, acid and salinity tolerance were measured. Inoculated cells were harvested by centrifugation at 12,000 rpm for 10 min at 4°C. After the supernatant was removed, the pellet was washed two times with 1X phosphate buffer (1X PBS). It was resuspended into 1 ml of PBS buffer adjusted to acidified solutions (up to pH 2.0 and 7.0) or salted solutions (7.5 and 15.0%). Cells were exposed to the acidic solution for 2.5 h and the high salt solution for 3.5 h at 37°C. The viable cell count was measured in order to assess cell survival, and it was calculated by the same method as thermal resistance.

### Bacterial Kinetics

The biomass was measured by dried cell weight. Cultured bacteria in 1,000 ml of MRS broth were centrifuged at 4,000 rpm for 10 min, washing twice, and then drying at 60°C for 3 days. All measurements were repeated three times. The concentration of glucose was measured by HPLC in the culture solution filtered through a 0.45-um membrane filter [HPLC machine: Dionex Ultimate 3000 (Thermo Dionex, United States/pump, autosampler, and oven), detector: Shodex RI-101 (Shodex, Japan), and column: Sugar-Pak (Waters, 300 × 6.5 mm, United States)]. Glucose (Junsei Chem, 98%) was used as glucose standard. Since batch culture was performed in an Erlenmeyer flask, the Monod equation was used to calculate the factors (Okpokwasili and Nweke, 2006).

### Statistical Analysis

All experiences were performed with three replications to check if there is any experimental bias except single strain selection from A001F8-72. Student’s *t*-test was used to compare assessments of thermal, acidic, and salinity tolerance. We considered a 5% significance level. The FDR correction method was used for multiple-testing issues.

### Whole-Genome Sequencing

Whole-genome sequencing was served by Macrogen Inc., using SMRT sequencing. Samples were prepared according to a guide for preparing SMRTbell template for PacBio sequencing. NanoDrop spectrophotometer (Thermo Fisher Scientific, Waltham, MA, United States) and PicoGreen quantified the concentration of gDNA. All samples passed screening QC criteria. For PacBio Sequel sequencing, 5 μg of gDNA was served for 10-kb library preparation. For gDNA less than 17 kb, the actual size distribution was evaluated by 2100 Bioanalyzer (Agilent). Sheared gDNA using g-TUBE (Covaris Inc., Woburn, MA, United States) was purified by AMPure PB magnetic beads (Beckman Coulter Inc., Brea, CA, United States) if the apparent size was greater than 40 kb. A total of 10-μl library was arranged using PacBio DNA Template Prep Kit 1.0. SMRTbell templates were annealed using Sequel Binding and Internal Ctrl Kit 3.0. The Sequel Sequencing Kit 3.0 and SMRT cells 1M v3 Tray were conducted for sequencing. The PacBio Sequel platform captured SMRT cells (Pacific Biosciences, Menlo Park, CA, United States). The next steps were followed as the PacBio Sample Net-Shared Protocol. Raw data from PacBio RS II was assembled by PacBio SMRT portal system and HGAP4 tool assembled. Genome assembly was conducted with genome size parameter set to 3 Mb. Assembled contig with low quality such as a short length (<20,000 bp) and low coverage (<50×) was eliminated. To correct assembly errors in the assembled genome sequence, the polishing process was repetitively conducted with a quiver algorithm until genomic variants were not found. Assembled genome was circularized by Circlator (Hunt et al., 2015).

### Annotation of Genomic Information

The genomes of *L. acidophilus* EG004 and EG008 strains were annotated, and genes were categorized by protein functions using Rapid Annotations using Subsystems Technology (RAST) server with version 2.0 (Aziz et al., 2008). To identify its functionality and safety as a probiotic, several genetic factors were detected. Antibiotic-resistant genes were inspected by NCBI BLAST with ARG-ANNOT and CARD databases (Gupta et al., 2014; Jia et al., 2016). Virulence factor and prophage gene were identified by VirulenceFinder 2.0 (Joensen et al., 2014) and PHASTER (Arndt et al., 2016), respectively. IslandView4 discovered genomic islands (Bertelli et al., 2017). Identification of bacteriocin was carried out using BAGEL4 (van Heel et al., 2018). To detect variants in the EG008 strain, a comparative analysis was conducted. To find a singleton in each strain, orthologous gene and singleton definition were conducted with OrthoVenn2 software (Xu et al., 2019). Identified singletons were double checked by the NCBI BLAST. Single-nucleotide variants were detected by nucmer including MUMmer 3.23 (Kurtz et al., 2004).

### Comparative Genomic Analysis With *Lactobacillaceae* Family

To identify closeness between our strains and the *Lactobacillaceae* family, 22 genomes were used and compared with EG004 and EG008 strains. Twenty-two genomes of *Lactobacillaceae* and a genome of *B. subtilis* were downloaded from the NCBI website. The genome of *B. subtilis* was used as an outgroup to make the phylogenetic root of *Lactobacillaceae* family. Average nucleotide identity (ANI) was calculated by JSpecies1.2.1 (Richter and Rosselló-Móra, 2009). The 16S rRNA sequences were investigated by RNAmmer and aligned by ClustalW2.1 (Lagesen et al., 2007; Larkin et al., 2007). The phylogenetic trees were generated by MEGA X with 1,000 bootstrap using the neighbor-joining method (Kumar et al., 2018). To compare functional gene contents, protein prediction of 22 *Lactobacillaceae* genomes was performed by the RAST server. We certified whether detected variants in EG008 strain existed in other *Lactobacillaceae*. In the case of genic single-nucleotide polymorphism (SNP), the gene including SNP was used as a blast query for comparison. In the case of intergenic SNP, a sequence with a length of 301 base pairs including the variant was used. Multiple-sequence alignment by ClustalW2.1 was used to confirm sequence detection. In comparison to singleton, BLAST was utilized, and identification of gene location was employed by Artemis (Rutherford et al., 2000). The pan-genome analysis was conducted using PGAP program with the cut-off filtering of *E*-value (<1e-10) (Zhao et al., 2012).

## Results

### Development of Heat-Resistant *L. acidophilus* Strain Based on Adaptive Laboratory Evolution Method

We isolated *L. acidophilus* EG004 strain from fermented dairy food and identified it using 16S rRNA sequencing (Supplementary Figure 1). First, in an experiment to find out the limit of heat resistance of the wild-type strain, we found out that it was unculturable at 75°C for 99 generations. To overcome this limit of heat resistance, we developed the EG008 *L. acidophilus* strain by applying ALE with different temperatures from 60°C to 72°C (Figure 1). After the application of the ALE method, preliminary heat screening was conducted to select strains with identical genotype that can significantly improve heat resistance in the wild-type population. As a result, *L. acidophilus* cultured in EG008 colony showed the highest possible improvement in heat resistance (47.768%) than others (Table 1).

**TABLE 1:** Results of preliminary investigations on heat resistance improvement for a single strain of wild-type *L. acidophilus* A001F8-72.

**Colony**	**Before (CFU/ml)**	**After heat shock (CFU/ml)**	**Survival rate (%)**	**SD**
A001F8-72-1	4.10e+08	1.67e+08	40.899	0.036
A001F8-72-2	4.57e+08	1.13e+08	25.213	0.065
A001F8-72-3	5.17e+08	1.47e+08	28.855	0.034
A001F8-72-4	3.47e+08	1.65e+08	47.768	0.014
A001F8-72-5	4.67e+08	1.81e+08	39.413	0.058

**FIGURE 2:**
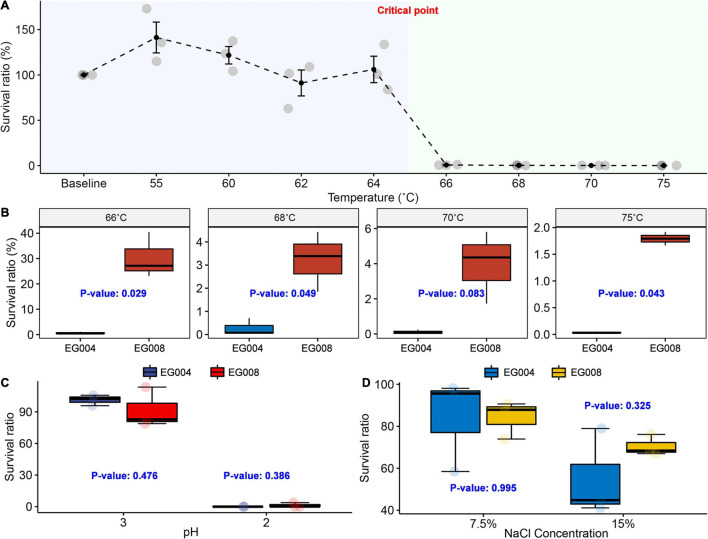
Experiment to confirm improved thermal resistance at critical temperature above 66°C. **(A)** Survival curve of *L. acidophilus* EG004 strain under different temperature conditions. The survival rate was calculated after 1-min exposure at each condition, and three repeated trials were performed. At 66°C, a sharp drop in survival was observed. **(B)** The newly developed *L. acidophilus* EG008 strain showed an improved survival ratio compared to the EG004 at critical point above 66°C. *P*-value is the result of the two-group *t*-test. **(C)** Investigation of survival rate in acidic conditions. **(D)** Measurement result of change in survival rate according to salt concentration.

*The survival rate (%) was calculated by repeating the preliminary heat resistance improvement experiment three times from each colony containing a single strain. In general, L. acidophilus has a sharp decrease in cell viability at a high temperature of 65°C or higher. This phenomenon was observed in our experimental results (Figure 2A). The evaluation was conducted at 66°C, where the survival rate rapidly decreased at.*

### Overcoming the Limit of Thermal Resistance of *L. acidophilus* EG004 Strain

As a result of conducting the experiment to determine the limit of heat resistance of the *L. acidophilus* EG004 strain, a significant decrease in survival rate was observed at 66°C (Figure 2A). We hypothesized that the *L. acidophilus* EG008 strain developed by the ALE method would have a significantly improved survival rate at such thermal limits. In the case of the newly developed *L. acidophilus* EG008 strain, the survival rate decreased as the temperature exceeded 66°C, but a statistically significant improvement in the survival rate was observed compared to the EG004 strain at a 5% significance level (Figure 2B). In particular, at 66°C, the identified limit of heat resistance of *L. acidophilus* EG004 strain, an average of 39.11% improvement in survival rate was observed (*P* = 0.029). At temperatures above 68°C, the tendency to decrease with a single-digit survival rate was similar to that of EG004, but a statistically significant improvement pattern of heat resistance was confirmed.

In previous studies that have developed heat-resistant strains in diverse species, it has been reported that the developed heat-resistant strain is additionally endowed with resistance to other types of stress such as acid and high salt concentration simultaneously (Min et al., 2020). Based on this fact, we expected that the newly developed *L. acidophilus* EG008 strain, which was endowed with improved thermal resistance, may also have cross-resistance. However, no statistically significant improvement in survival rate was observed under strongly acidic conditions (Figure 2C) and salt concentration (Figure 2D), which suggests that genes related to heat resistance could be independent of genes related to acid and salt resistance in *L. acidophilus*. In addition, in order to verify whether the probiotic functionality and fermentation performance were maintained, a comparative evaluation was performed with *Lacticaseibacillus rhamnosus* GG (LGG), which has well-proven functionality. As a result, the two experimental strains showed higher acid resistance compared to the LGG stain, and the salt-resistance and bile salt resistance were similar (Supplementary Figure 4). In an assessment of fermentation performance, all estimated summary statistics of both strains were similar (Supplementary Table 2).

### Complete Genomic Analysis for *L. acidophilus* EG004 and EG008 Strains

**FIGURE 3:**
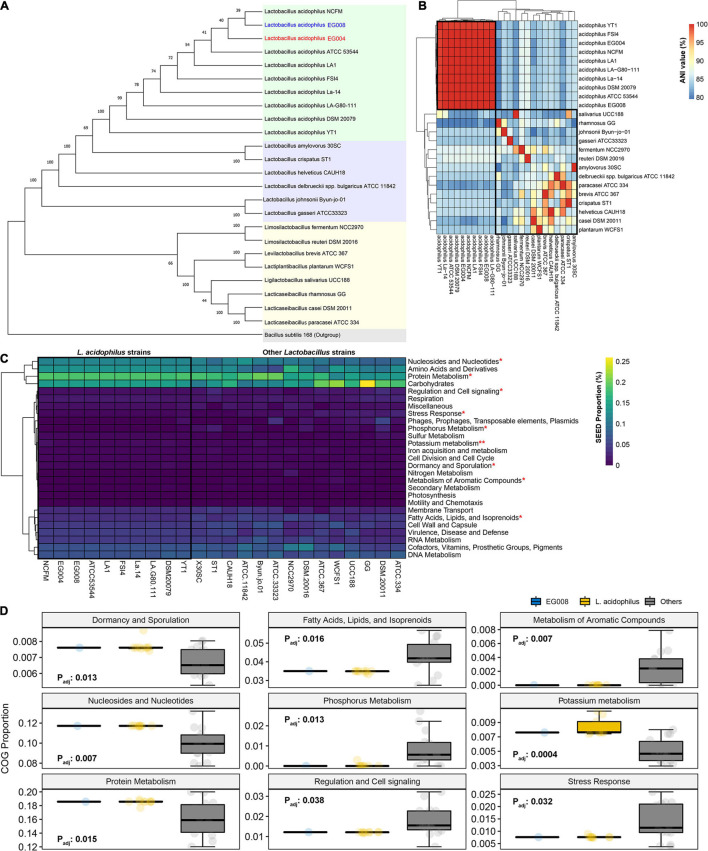
A comparative genome analysis for *Lactobacillaceae* based on the complete genome sequence of the newly developed *L. acidophilus* strain. **(A)** Neighbor-joining tree based on 16S rRNA sequences for 24 *Lactobacillaceae* families including the strain newly developed in this study. Bootstrapping was performed 1,000 times, and *Bacillus subtilis* 168 was considered as an outgroup. **(B)** Hierarchical clustering results based on average nucleotide identity among 24 *Lactobacillaceae* families. **(C)** A comparison of predictive SEED ratios between 24 *Lactobacillaceae* strains. **(D)** SEEDs with statistically significant differences between *L. acidophilus* and the rest of the *Lactobacillaceae* strains at 5% significance level after multiple testing adjustments. *P < 0.01 and **P < 0.001 in comparison of *L. acidophilus* and other strains.

We completed the whole genome sequences from EG004 and EG008 strains through long-read sequencing technology (Supplementary Table 1) to further reveal genomic characteristics to confirm that the newly developed strain is *L. acidophilus*. Constructed phylogenetic tree based on the 16S rRNA from 22 publicly available *Lactobacillaceae* whole-genome sequences confirmed that the developed strains were one of the *L. acidophilus* species (Figure 3A). Of a total of 24 *Lactobacillaceae* families, full-length sequences of 10 *L. acidophilus* strains used in the analysis showed a very high degree of similarity to each other (avg. 99.835%) and relatively low similarity (avg. 93.992%) to other *Lactobacillaceae* families (Figure 3B). This result confirms once again that the heat-resistant strain we developed is *L. acidophilus* and suggests that strains of *L. acidophilus* species have a specific genetic background in common, which is distinct from other *Lactobacillaceae* families. Likewise, in the functional comparison based on gene annotation, no significant differences were observed between *L. acidophilus* strains (Figure 3C), but statistically significant differences were found in nine SEED terms at 5% significance level (Figure 3D) between *L. acidophilus* and other *Lactobacillaceae*. These results are ideal considering that our secondary goal is to maximize heat resistance while maintaining desirable properties such as the antimicrobial properties of *L. acidophilus*. We confirmed that the genome of the *L. acidophilus* EG008 strain retained various antibacterial-related genes such as bacteriocin (Supplementary Figure 2). Furthermore, examining the features of the full-length genome, seven genomic islands, and two prophage regions were found identically in both EG004 and EG008 strains (Supplementary Figure 3). In addition, the pan and core genome analysis revealed that both strains are open pan-genome. Antibiotic resistance-related genes and a virulence factor were not found in both genomes. These results are one of the pieces of evidence showing that the newly developed EG008 strain has improved heat resistance while maintaining the basic functional advantages of the existing wild-type *L. acidophilus*.

### Potential Genetic Factor Related to Heat Resistance Improvement Through Comparative Genome Analysis

**FIGURE 4:**
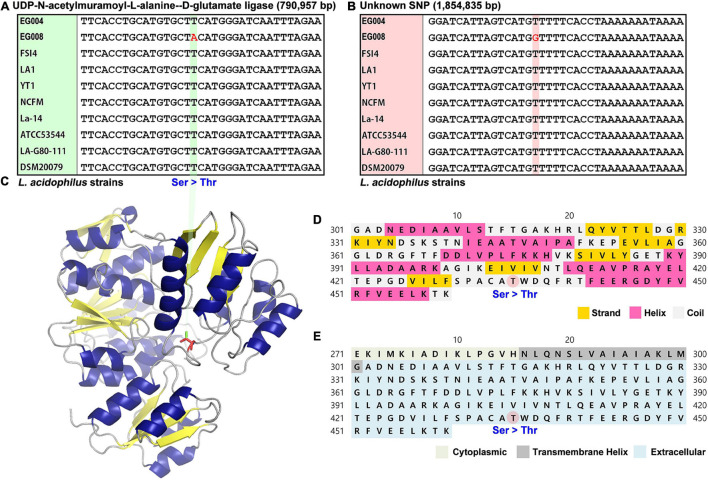
Two single-nucleotide polymorphisms (SNPs) were found by comparing the complete genome sequences of *L. acidophilus* EG004 and EG008 strains. **(A)** A non-synonymous SNP is located at 790,957 bp of genic region in EG008 strain. This genotype change causes the serine amino acid to be transformed into threonine. **(B)** A SNP found in the intergenic region of the EG008 strain. **(C)** Prediction of 3D structural change of MurD protein by the identified non-synonymous SNP. The serine-to-threonine changes trigger a change from hydroxyl to acyl group (red) in the side chain. **(D)** The 435th amino acid of the secondary structure is predicted to be the coli part by PSIPRED tool. **(E)** It is the part of the coil that connects the beta sheet of the third domain and the alpha helix, which is located on the extracellular matrix.

We hypothesized that the genetic factors to improving heat resistance could be found by comparing the whole genome sequences of EG004 and EG008 strains. Interestingly, only two nucleotides were different across the two genomes. As a result of performing multiple-sequence alignment including other *L. acidophilus* strains whose full-length genome sequences were published, a different genotype of these two SNPs was observed only in our newly developed EG008 strain. One of the two SNPs is located in the murD gene, which synthesizes UDP-N-acetylmuramoyl-L-alanine–D-glutamate ligase (Figure 4A), while the other SNP is located intergenically between the galT gene (1,854,039–1,854,833 bp) and the IdtD gene (1,854,986–1,856,194 bp) (Figure 4B). Both mutations may be strong candidates for conferring heat resistance, but we further investigated for non-synonymous SNPs that are easy to be interpreted biologically. A variant in the murD gene of *L. acidophilus* EG008 strain causes substitution of nucleotide from T (thymine) to A (adenine). Subsequently, this substitution induces changes in amino acid from S (serine) to T (threonine) at 435th amino acid residue of UDP-N-acetylmuramoyl-L-alanine–D-glutamate ligase (Figure 4C). Through a protein structure analysis, we found that this substitution can trigger a change from hydroxyl to acyl group in the coil part of the protein (Figure 4D), which is located in the extracellular matrix (Figure 4E). These results provide an insight into the mechanism of *L. acidophilus* strains with improved heat resistance at the molecular biology level.

## Discussion

In this study, we achieved the primary goal for improving the thermal resistance at 65°C or more to secure the ease of industrial use of the *L. acidophilus* strain through stepwise ALE method (Figure 1). We have demonstrated that the newly developed *L. acidophilus* EG008 strain had better thermal resistance than wild type at high temperatures of 65–75°C (Figure 2B). Recently, studies on the development of EG008 strains using ALE have been conducted (Kulkarni et al., 2018; Min et al., 2020), but most of the experiments were performed at temperatures below 65°C to ensure applicability in LTST pasteurization (Vaudagna et al., 2002). Therefore, the EG008 strain developed in this study is expected to be utilized for HTST pasteurization, which sterilizes at a relatively high temperature.

It is well known that repetitive heat stimulation employed in the ALE process can also induce resistance to other stresses such as acid or osmotic pressure by changing fatty acid composition of the cell wall, which is termed as cross protection (Kim et al., 2001; Meena et al., 2016). Based on this fact, we expected that the *L. acidophilus* EG008 strain would exhibit the same phenomenon as well as a significant improvement in heat resistance. However, contrary to expectations, no cross-protection effects were observed, and only thermal resistance was found to be improved (Figures 2C,D). This suggests that the increased heat resistance may be due to reasons other than changes in fatty acid composition. Another possibility is that the three biologically replicated samples were not quantitatively sufficient in their sample size to confirm such difference in cross resistance, which can be a limitation of the study. Further research will be needed to elucidate the mechanisms for the stress adaptation in the strain.

The second goal of this study was to maintain the beneficial functions of the existing *L. acidophilus* wild type as much as possible while increasing heat resistance. To investigate this, we constructed complete genome sequences of the developed (EG008) and the wild-type (EG004) strains using third-generation sequencing technology and performed a comparative genome analysis. As a result, only two SNPs were found (Figures 4A,B) between the sequences (Figures 3A,B). In addition, there was no difference between the two strains in the gene annotation and functional analysis, suggesting that the characteristics of the *L. acidophilus* EG004 are maintained. We found three regions encoding bacteriocin, acidocin, enterolysin A, and helveticin J, in both genomes, which is an important feature for probiotic efficacy. Bacteriocin is a proteinaceous or peptidic toxin secreted by bacteria with antibacterial activity against other strains except for itself (Riley, 1998; Gálvez et al., 2007). It is well known for its function in the gastrointestinal tract to inhibit the invasion of pathogens or competitors and affect the host’s immune system (Dobson et al., 2012; Hegarty et al., 2016). As these substances are widely used as biological preservatives due to their high stability in animals including humans, we believe the ability to produce bacteriocin is considered a beneficial property for industrial use of probiotics.

We found two SNPs in the genome of EG008 strain, one of which was a non-synonymous SNP located in murD gene encoding UDP-N-acetylmuramoyl-L-alanine–D-glutamate ligase (Figure 4A). This enzyme is involved in the synthesis of peptidoglycan, a component that strengthens the bacterial cell wall (Bertrand et al., 1997). When the synthesis of this enzyme is not performed normally or the integrity of the enzyme is destroyed, the cell is dissolved by the turgor pressure inside the cell. Previous studies have shown that the expression of this protein increased when heat stimulation was applied in various strains such as *Staphylococcus aureus* and *Streptococcus thermophilus* (Mengin-Lecreulx et al., 1999; Li et al., 2011). This suggests that the expression of UDP-N-acetylmuramoyl-L-alanine–D-glutamate ligase could be the defense mechanism of bacteria against external heat stress. The non-synonymous SNP found in our results changes the 435th amino acid residue of this enzyme from serine to threonine, causing hydrogen loss and acyl gain in the side chain (Figures 4A,C). We suspected that this change caused a number of changes, such as the volume of the molecule and the location of the hydroxyl groups, affecting the three-dimensional structure and hydrophilicity, thereby altering the resistance to thermal stimulation. In addition, we confirmed that the genotype of these SNPs was specifically found only in *L. acidophilus* EG008 strain (Figures 4A,B). This is one of the evidences that these two SNPs, artificially evolved by the ALE method, can be associated with the improved thermal adaptability.

Another SNP was found in the non-coding region, and there were two genes nearby this SNP (Figure 4B). One of them was a gene encoding galactose-1-phosphate uridylyltransferase, which is 2-bp away from the SNP located between the core promoter and the ORF starting point. The other was a gene that synthesizes L,D-transpeptidase. This peptidase uses peptidoglycan or its precursor as a substrate to form 3-3 peptidoglycan crosslinks in Gram-positive bacteria (Gupta et al., 2010; Peltier et al., 2011). Therefore, there may be mechanisms to regulate the rigidity of the bacterial cell wall by regulating the level of expression of this gene (Brammer et al., 2015). It is also known that L,D-transpeptidase enhances the synthesis of (p)ppGpp alarmone (Hugonnet et al., 2016). The ppGpp induces a stringent response that inhibits RNA synthesis in emergency situations such as heat stress and amino acid shortage (Jain et al., 2006). It is known to be a gene involved in the Cpx stress response, one of the well-known envelope damage systems in *E. coli*, and is upregulated with YgaU when subjected to external stress such as high temperature or high osmotic pressure (Bernal-Cabas et al., 2015; Ultee et al., 2019). Based on these evidences, although it is an intergenic variant, there may be a mechanism that affects the expression level of adjacent genes and ultimately contributes to imparting heat resistance.

All of the SNPs found in this study were related to cell wall synthesis, specifically the peptidoglycan layer. The cell wall maintains the bacterial cell structure, is responsible for the movement of substances, and interacts with host cells or pathogens (Sequeira et al., 1977; Sengupta et al., 2013). Thus, since the cell wall is directly affected by various environmental stresses, including thermal stress, the evolutionary response to external stimuli may take precedence. Evidence for this speculation can be found in previous studies investigating changes in cell walls when external physical stresses such as pH, temperature, osmotic pressure, and high bile salt concentration are applied in *L. acidophilus* (Khaleghi et al., 2010; Grosu-Tudor et al., 2016; Palomino et al., 2016). Putting all of this together, it indicates that EG008 strain may have evolved to survive at higher temperatures by making cell walls more rigid.

This study has some practical limitations. The first experimental limitation is that the heat-adapted strain selected after ALE treatment may not be the population of strains with the best heat resistance. This occurs by physical restriction in the screening process to isolate single colonies from the population generated through ALE. There are about 10^9^ CFUs randomly mutated strains in the population generated after ALE treatment, but it was not possible to separate the number of all cases into single colonies due to the loss in the dilution process and experimental limitations on culturable colonies on the plate. As a second limitation, although the whole genome sequences were generated using PacBio long-read sequencing technology, the technical limitation for genotyping error still remained. However, the whole genome we completed had depth coverage of 341.59X for the EG008 strain and 255.60X for the EG004 strain, and the possibility of genotyping errors was slim. Thirdly, gene expression analysis was not considered for the comparison of wild-type and heat-adapted strains. Since the genetic variation found in this study can affect the expression level of the transcript, it is expected that this fact will be further revealed if a comparison of the whole transcriptome through RNA sequencing is conducted in the near future. Finally, this study did not cover the experimental validation of the two SNP candidates that were supposed to confer heat resistance to the *L. acidophilus* EG008 strain. Although many gene manipulations using the CRISPR/cas9 system have been reported, many technical difficulties remain in applying CRISPR/cas9 technology to gram-positive bacteria such as LAB (Leenay et al., 2019). It is our ultimate goal to experimentally verify the mutations detected after ALE and to reveal a direct relationship between phenotypes and genetic factors. We expect that, in the near future, if technologies such as genetic scissors for microorganisms become common, the results of this study can be verified.

Despite these technical limitations, we have succeeded in improving our primary target, the heat-resistant limit temperature of 75°C, to maximize the industrial usability of *L. acidophilus* strain. One step further, biomarkers associated with improved thermal resistance were identified through whole genomic analysis. We anticipate the *L. acidophilus* strain developed in this study to be directly helpful in industrial sites where a stronger heat-resistant bacterial strain is required. We also believe that the identified biomarkers provided insights into the mechanisms of heat resistance and evolution of bacteria, including *L. acidophilus*.

## Data Availability Statement

The datasets presented in this study can be found in online repositories. The names of the repository/repositories and accession number(s) can be found below: https://www.ncbi.nlm.nih.gov/, BioProject PRJNA657145.

## Author Contributions

MS and HBK designed the study. SJ and MS wrote the manuscript. SJ analyzed the data. SC generated sequencing data. HKK and YC reviewed and edited the manuscript. All authors contributed to the article and approved the submitted version.

## Conflict of Interest

SC and HBK were employed by the company eGnome, Inc. The remaining authors declare that the research was conducted in the absence of any commercial or financial relationships that could be construed as a potential conflict of interest.

## Publisher’s Note

All claims expressed in this article are solely those of the authors and do not necessarily represent those of their affiliated organizations, or those of the publisher, the editors and the reviewers. Any product that may be evaluated in this article, or claim that may be made by its manufacturer, is not guaranteed or endorsed by the publisher.
