# Supplementary material for: Complete Genome Sequence of the Newly Developed Lactobacillus acidophilus Strain With Improved Thermal Adaptability
Source: Front Microbiol. 2021 Sep 24;12:697351. doi: 10.3389/fmicb.2021.697351 (PMC8498822; doi:10.3389/fmicb.2021.697351)
Supplement: Supplementary file 1 [file Data_Sheet_1.docx]

**Supplementary Information**

**Contents**

**Supplementary Figures**

[Figure S1. Staining image of *L. acidophilus* EG004 and EG008 strains. 2](#_Toc78555271)

[Figure S2. Antibacterial-related genes found in the genome of *L. acidophilus* EG004 and EG008 strains 3](#_Toc78555272)

[Figure S3. Genomic features of *L. acidophilus* EG004 and EG008 strains. 4](#_Toc78555273)

[Figure S4. Physiological activities of *L. acidophilus* EG004 and EG008 strains. 5](#_Toc78555274)

**Supplementary Tables**

[Table S1. Assembly statistic for complete genome of *L. acidophilus* EG004 strain and EG008 strain. 6](#_Toc78555278)

[Table S2. Microbial kinetics for *L. acidophilus* EG004 and EG008 strains. 7](#_Toc78555279)

**Supplementary Figures**


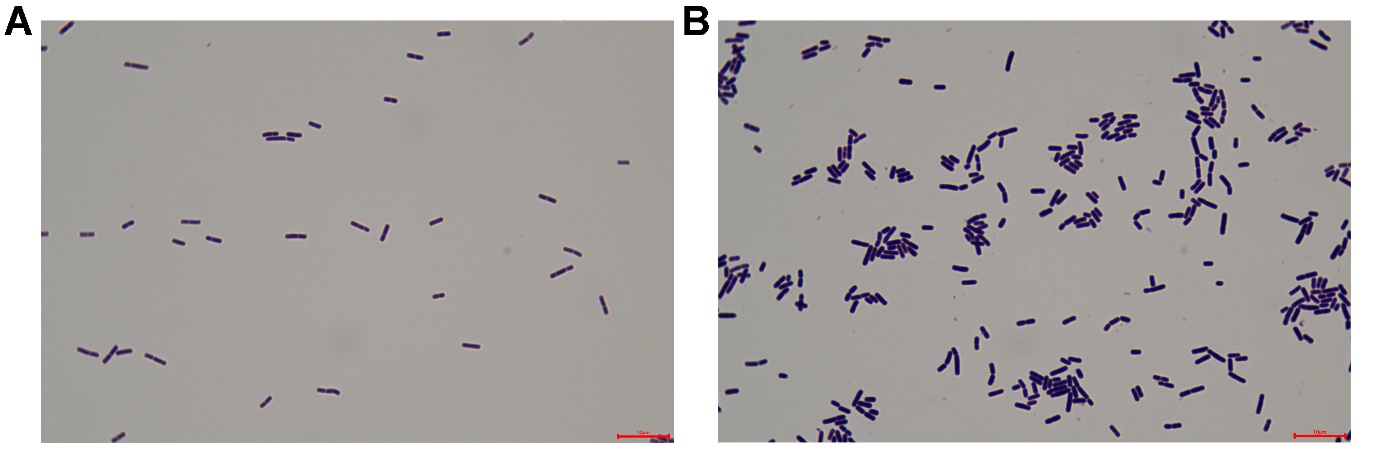


Figure S1. Staining image of *L. acidophilus* EG004 and EG008 strains.

We observed bacterial morphology of *L. acidophilus* **(A)** EG004 and **(B)** EG008 strains. After 16 hours of incubation, two strains were prepared for microscopic observation. Bacterial morphology was examined under a light microscope at 1000x magnification after gram staining. We verified that both strains are rod-shaped and gram-positive bacteria. The length of the red bar at the bottom represents 10 μm.


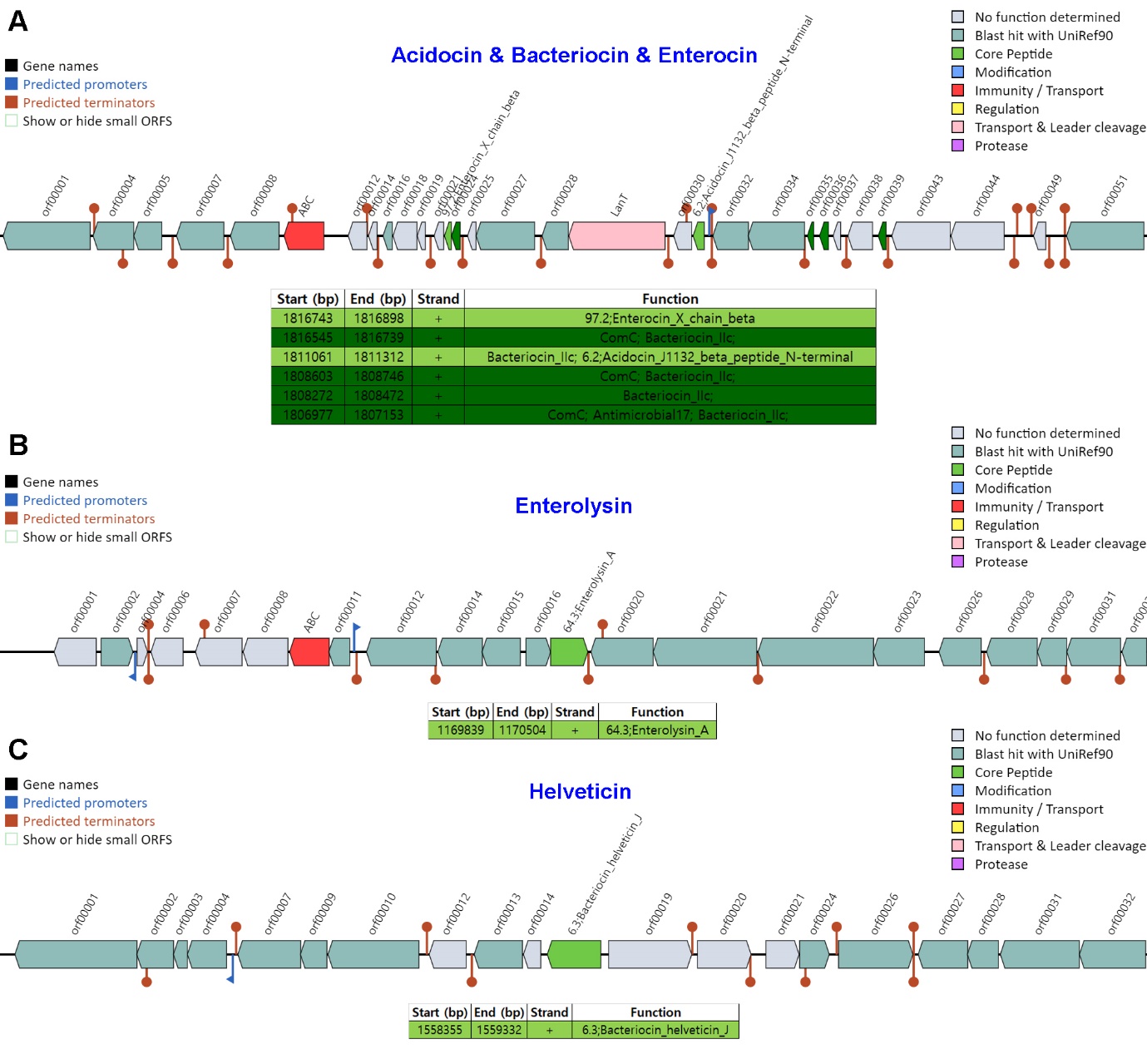


Figure S2. Antibacterial-related genes found in the genome of *L. acidophilus* EG004 and EG008 strains

We identified that all genes related to antibacterial action, such as Acidocin, Bacteriocin, Enterocin, Enterolysin, and Helveticin, are maintained on the genome of the *L. acidophilus* EG004 using BAGLE4 software. There were no differences between the EG004 and EG008 strains.


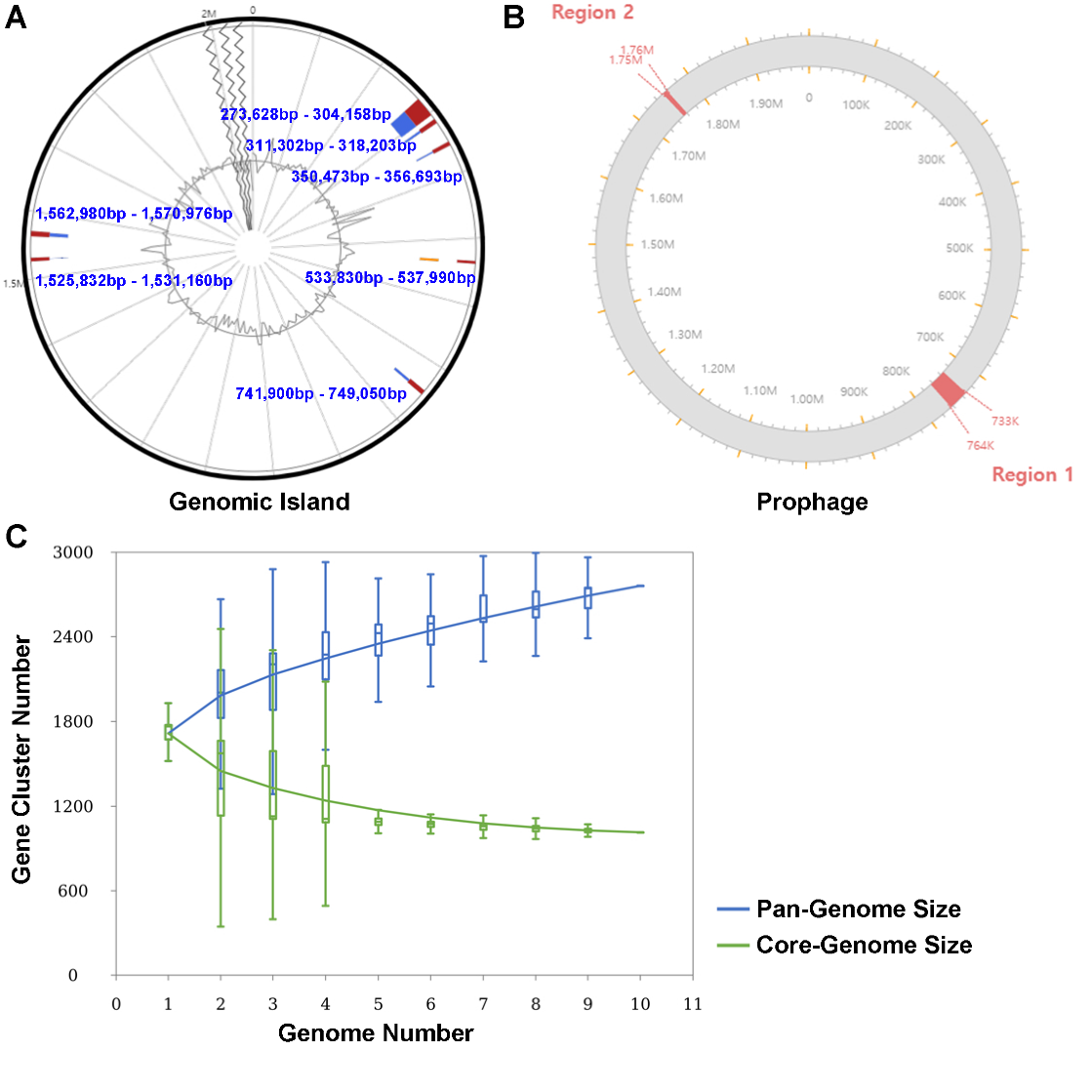


Figure S3. Genomic features of *L. acidophilus* EG004 and EG008 strains.

**(A)** Seven predicted genomic islands on the *L. acidophilus* EG004 and EG008 strains. **(B)** Two complete prophage regions identified by PHASTER tool. **(C)** Result of pan and core genome analysis. The results demonstrated that the pan-genome of *L. acidophilus* EG004 and EG008 strains is open, given the pattern of influx of new genes while the number of core genes is about half of that of the pan genes. There was no difference between EG004 and EG008 strains in all annotations.


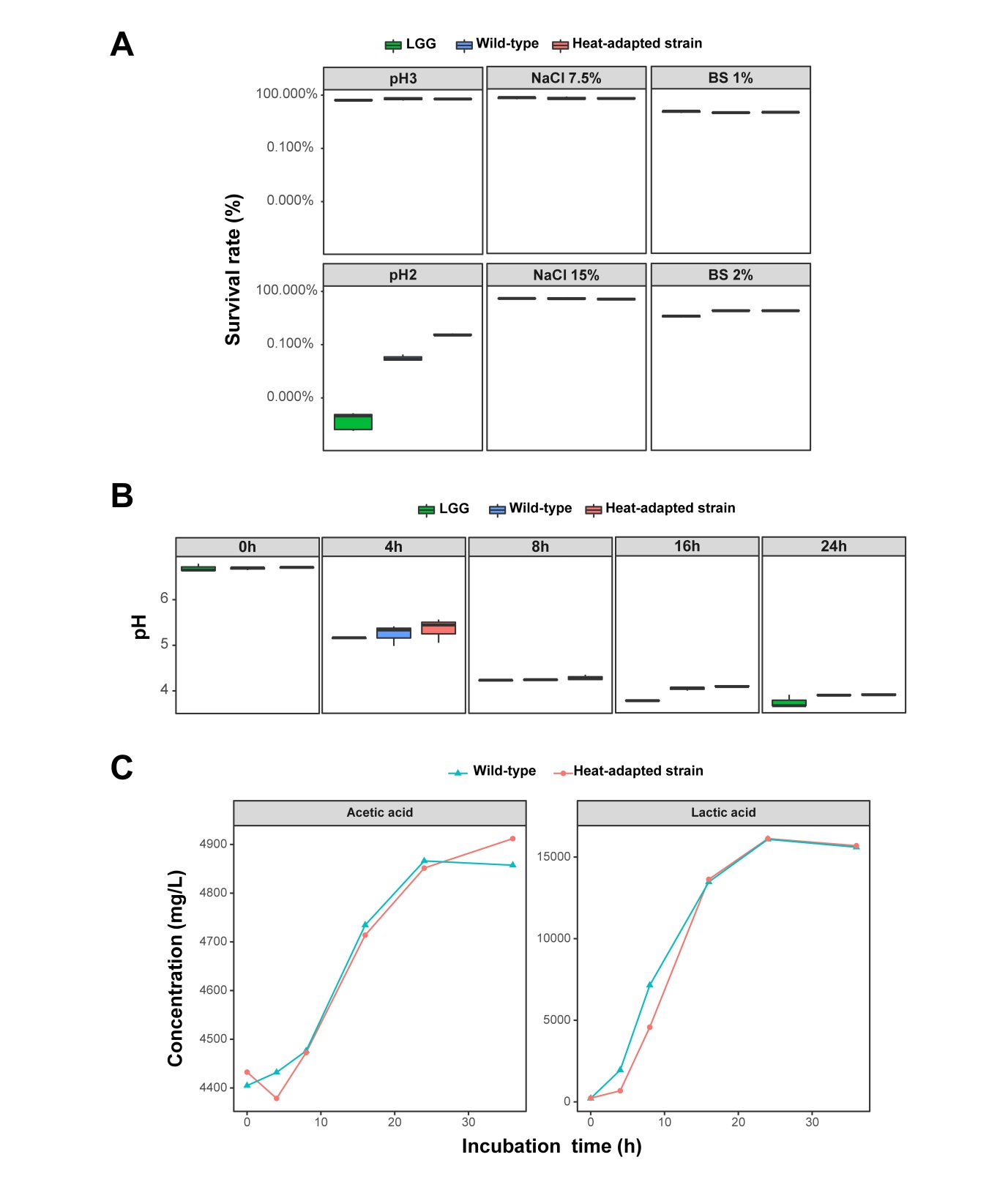


Figure S4. Physiological activities of *L. acidophilus* EG004 and EG008 strains.

**(A)** Resistance comparison to external stresses with LGG strain. Resistance was assessed to acid, salt, and bile salt stresses. **(B)** pH change according to bacterial culture time. **(C)** Changes in acetic acid and lactic acid concentration.

**Supplementary Tables**

Table S1. Assembly statistic for complete genome of *L. acidophilus* EG004 strain and EG008 strain.

| **Assembly statistic** | ***L. acidophilus* EG004** | ***L. acidophilus* EG008** |
| --- | --- | --- |
| # of subreads | 85,438 | 1,003,160 |
| Avg. alignment length | 5,000 | 3,388 |
| N50 of contig | 1,991,561 | 4,036 |
| Genome coverage | > 100x | > 100x |
| Maximum contig length (bp) | 1,991,559 | 2,001,616 |

Table S2. Microbial kinetics for *L. acidophilus* EG004 and EG008 strains.

| **Factor** | ***L. acidophilus* EG004** | ***L. acidophilus* EG008** |
| --- | --- | --- |
| μ_max_ (-h) | 0.0238 | 0.0237 |
| Y_X/S_ (cell/substrate) | 0.0911 | 0.0825 |
| t_d_ (h) | 29.08 | 29.31 |
| K_s_ (g/L) | 2.076 | 2.079 |
| q (g/h) | 0.2616 | 0.2866 |

μ: the specific growth rate of the bacteria, μ_max_: the maximum specific growth rate, S: the concentration of the limiting substrate for bacterial growth, Ks: the half-velocity constant, X: the total biomass, Y_X⁄S_: fermentation growth yield, t: cultural time, and q: substrate consumption ratio
